# Association of striking life events with primary breast cancer in 265 Chinese women: a case-control study

**DOI:** 10.18632/oncotarget.18744

**Published:** 2017-06-27

**Authors:** Yan Lin, Shengsheng Wang, Xiaohui Zhang, Changjun Wang, Guangliang Shan, Yidong Zhou, Feng Mao, Jinghong Guan, Xin Huang, Ying Zhong, Qiang Sun

**Affiliations:** ^1^ Department of Breast Disease, Peking Union Medical College Hospital, Peking Union Medical College, Beijing 100730, China; ^2^ Physical Examination Center, Peking Union Medical College Hospital, Peking Union Medical College, Beijing 100730, China; ^3^ Department of Epidemiology, Institute of Basic Medical Sciences, Peking Union Medical College, Beijing 100730, China

**Keywords:** breast cancer, case-control study, striking life events

## Abstract

The current case-control study investigated the relationship between striking life events and breast cancer in Chinese women. A total of 265 primary breast cancer patients and 265 controls were enrolled with matching for age and completed questionnaires. Logistic regression analysis was used. Thirty-nine breast cancer patients reported striking life events and twenty-four of the controls reported striking life events. The number of striking life events was significantly greater in patients (*p* = 0.04). It indicated a striking life event led to a 1.726-fold increased HR (95% CI 1.005-2.965, *p* < 0.05) for breast cancer with adjustment for age, and a 1.811-fold increased HR (95% CI 1.021 - 3.212, *P* < 0.05) with adjustment for age, BMI, and late age at menopause. High BMI (OR: 1.680, 95% CI: 1.258-2.196, *p* < 0.05) and a family history of breast cancer (OR: 2.244, 95% CI: 1.065-4.729, *p* < 0.05, respectively) increased the risk of breast cancer, and late age at menopause decreased the risk for breast cancer (OR: 0.513, 95% CI: 0.303-0.868, *p* < 0.05). Our findings indicate a significant association between striking life events and breast cancer in Chinese women, especially in the subpopulation with high BMI or family history of breast cancer.

## INTRODUCTION

Breast cancer has a high morbidity and mortality throughout the world, accounts for 23% of all malignant tumors, and is a major cause of cancer deaths among women [1]. The number of established and hypothesized risk factors for breast cancer exceeds that of any other cancer, but there are conflicting reports on these risk factors. Our understanding of factors that increase the risk of breast cancer, as well as its biology and molecular basis, has greatly increased in recent decades. Recent studies have shown that genetic, physiological, and behavioral factors can increase the risk of breast cancer, including early menarche, late menopause, nulliparity or bearing a first child at a late age, overweight after menopause, certain types of benign breast diseases, alcohol consumption, and long-term use of menopausal estrogen replacement therapy [2–5].

Previous research has placed greater emphasis on certain of these traditional risk factors. Remarkably, few studies have examined the role of psychological factors on breast cancer. These psychological factors include personality traits, behavioral characteristics, and the presence of psychological stress, crucial life events, and social support. Very little research has examined the potential effect of striking life events on breast cancer. A striking life event is defined as an event that can lead to an acute stress disorder or acute anxiety disorder, conditions characterized by adverse and anguishing experiences and physiological responses. Striking life events include a change in marital status, such as separation, divorce, or widowhood; death of a spouse, child, or close relative; illness of friend; personal health problems; and change in financial status [6–9]. In 1893, Snow et al. first described the relationship between psychologically striking events and breast cancer. They studied 250 breast cancer patients, 156 of whom experienced the death of relatives (a “major life event”). The short-term or acute psychological response to a striking life event differs from chronic stress and depression, and has become a matter of increasing concern.

A more recent prospective cohort study found that chronic stress in women was associated with an increased incidence of breast cancer, possibly due to inhibition of estrogen synthesis [10]. Based on a meta-analysis of 7 studies that included 99,807 women, we previously found that women with striking life events had a 1.5-fold greater risk of breast cancer, and women with severe striking life events had a 2-fold greater risk [6]. Although this previous research showed a possible positive association between striking life events and primary breast cancer, other research indicated no unified opinion regarding the correlation between short-term exposure to stressful life events and primary breast cancer [11]. In fact, some clinical studies demonstrated positive evidence [12–15], and others indicated no evidence of this correlation [16–19], the tenor of the thoughts reflected the divergence in the priorities of the two sides, which carried out more prospective studies [20–26]. Due to the convergent evidence, an integrative approach might be advantageous in terms of sample size of population, setting suitable priorities in questionnaire content, and improving the methods with a more complete and less biased picture.

The differences in these previous studies may be due to differences in the ways of thinking, life style, culture, and philosophy between Eastern and Western women. For example, in Western societies, anxiety was more commonly reported than in non-Western societies. About ten percent of people in North America and Europe were experiencing clinical anxiety compared to approximately eight percent in the Middle East and six percent in Asia. The opposite was true for depression, depression was the lowest in America but highest in certain areas of Asia [42]. It seems that acute stress may cause by different factors in Eastern and Western women, so the role of psychologically striking events in Chinese women may be unique. In fact, no clinical study has yet examined the association of short-term exposure to stressful life events on the incidence of primary breast cancer in Chinese women. Regarding that few psychological factors and breast cancer risk studies have been done for Eastern populations, we examined the effect of striking life events on breast cancer in Chinese women, with use of multivariable analysis to control for possible confounding in the present study.

## RESULTS

### Basic characteristic of cases and controls

We enrolled 265 patients with breast cancer and 265 age-matched controls, all of whom completed the questionnaire and were profiled (Table 1). Due to the use of age-matching, these groups had similar numbers of individuals in each age category. The highest incidence of breast cancer was in women aged 40 to 49 years-old (44.5%), followed by those who were 50 to 59 years-old (28.7%). Thus, the age of the highest incidence of breast cancer in China is younger than in Western countries. Early menarche was more common in the controls, in agreement with our previous report [27]. Analysis of BMI indicated that overweight and obesity were significantly more common in cases than controls. Interestingly, more cases than controls had a family history of breast cancer. Menopausal status and use of oral contraceptives were similar in cases and controls. Thus, patients with breast cancer were significantly more likely to have a family history of breast cancer, higher BMI, and late menarche and controls were significantly more likely to have low or no stress.

**Table 1:** Basic characteristics of women with breast cancer (cases, *n* = 265) and without breast cancer (controls, *n* = 265)

Characteristic	Cases (*n*, %)	Controls (*n*, %)	*P* value^*^
Age (years)			
20-29	4 (1.51)	3 (1.13)	0.7055
30-39	43 (16.23)	58 (21.89)	0.1356
40-49	118 (44.53)	117 (44.15)	0.9480
50-59	76 (28.68)	68 (25.66)	0.5050
60-70	24 (9.06)	19 (7.17)	0.4458
Menarche (years)			
<13	61 (23.2)	85 (32.8)	0.0470
13-14	68 (25.66)	60 (22.64)	0.4795
>14	136 (51.32)	120 (45.28)	0.3173
BMI (kg/m^2^)			
<18.5	13 (4.91)	12 (4.53)	0.8415
18.5-24	120 (45.25)	170 (64.15)	0.0033
24-28	105 (39.62)	71 (26.79)	0.0104
≥28	27 (10.19)	12 (4.53)	0.0163
Stress score			
0	19 (7.17%)	35 (13.21%)	0.0295
1-3	62 (23.4%)	54 (20.38%)	0.4576
4-6	142 (53.58%)	129 (48.68%)	0.4297
7-9	42 (15.85%)	47 (17.74%)	0.5961
Breast operation history			
With	41 (15.47)	36 (13.58)	0.5688
Without	224 (84.53)	229 (86.42)	0.8143
Breast cancer history			
With	25 (9.43)	12 (4.53)	0.0326
Without	240 (90.57)	253 (95.47)	0.5582
OCP			
With	11 (4.15)	10 (3.77)	0.8273
Without	254 (95.85)	255 (96.23)	0.9646
Post-menopausal			
Yes	53 (20.0)	62 (23.4)	0.4013
No	212 (80.0)	203 (76.6)	0.6586

Abbreviations: BMI, body mass index; OCP, use of oral contraceptive pills more than 1 year; Breast cancer history, refers to a family of relatives (parents, children, brothers, or sisters) with breast cancer; Breast operation history, It does not include a biopsy before radical surgery for breast cancer in cases group.

*Chi-square test.

Among the 265 cases with breast cancer, 39 patients reported striking events in the 3 years before the onset of breast cancer, 7 cases reported 2 striking events, and no cases reported more than 2 striking events. Eight of the patients had striking events but did not provide detailed descriptions; however, after receiving explanations from the researchers, these 8 patients classified their events as related to feelings (including divorce, separation, separation, illegal love affair), economics (including unemployment, housing disputes, major property losses, early retirement), or family (including serious illness or death of family member, death of a close friend, imprisonment of a relative, being away from relatives).

### Striking life events in cases and controls

The overall incidence of striking life events was only 17.4% among cases and 9% among controls. Table 2 shows the occurrence of different types of striking events during the previous 3 years in cases and controls. Striking life events related to relatives or friends were most common in both groups, followed by events related to emotions or marriage, and events related to money, work, or studies. There were no significant differences between cases and controls in the different types of striking life events. However, there were significantly more total striking life events in cases than in controls (*p* = 0.0441).

**Table 2:** Presence of different types of striking life events in women with breast cancer (cases) and without breast cancer (controls)

Type of striking event	Cases (*n,* %)	Controls (*n,* %)	*P* value*
Money, work, or study	7 (2.64)	4 (1.51)	0.3657
Illness or death of friend/relative	24 (9.06)	14 (5.28)	0.1048
Emotions and marriage	8 (3.02)	6 (2.26)	0.5931
Total number of women	39 (14.72)	24 (9.06)	0.0609
Total number of events	46 (17.35)	25 (9.43)	0.0441

*Chi-square test

### Multivariable logistic regression analysis of continuous variables

Table 3 shows the results of multivariate regression analysis of factors related to breast cancer, with potential risk factors treated as continuous variables. High BMI (HR: 1.680, 95% CI: 1.258-2.196, *p* = 0.0001) and breast cancer in a first-degree relative (HR: 2.241, 95% CI: 1.065-4.729, *p* = 0.0335) were strongly associated with breast cancer. Interestingly, post-menopausal status was a protective factor (HR: 0.513, 95% CI: 0.303-0.868, *p* = 0.0129). Analysis of the relationship between striking life events and breast cancer indicated a marginally insignificant association (HR: 1.715, 95% CI: 0.976-3.017, *p* = 0.0609).

**Table 3:** Multivariate logistic regression analysis of the relationship of continuous variables with breast cancer

Risk factor	OR	95% CI	*P* value
Age	1.237	0.961 - 1.593	0.0990
BMI	1.680	1.258 - 2.196	0.0001
Age at menarche	1.237	0.999 - 1.531	0.0510
Stress	1.196	0.963 - 1.485	0.1047
Breast operation history	1.072	0.644 - 1.785	0.7897
Breast cancer history	2.244	1.065 - 4.729	0.0335
OCP	1.071	0.424 - 2.706	0.8847
Post-menopausal	0.513	0.303-0.868	0.0129
Striking life event	1.715	0.976-3.017	0.0609

Abbreviations: OR, Odds Ratio; CI, confidence interval of estimated HR; OCP, use of oral contraceptive pills more than 1 year.

**Figure 1:**
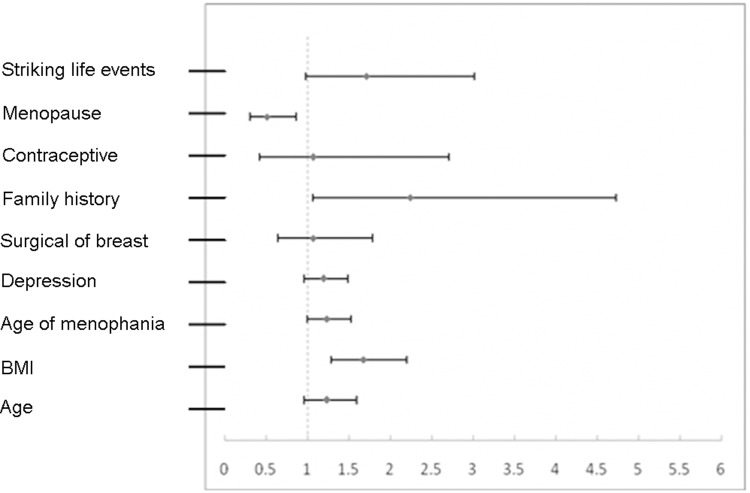
Forest plot of factors associated with breast cancer based on analysis of continuous variables

Forest plots (Figure 1) show these same data graphically, and emphasize that only post-menopausal status was protective, and that only a family history of breast cancer and elevated BMI were significantly harmful.

### Multivariable logistic regression analysis of rank variables

Analysis of rank variables rather than continuous variables may yield different results. Thus, we re-analyzed these data by use of multivariable logistic regression of rank variables (Table 4). These results were in general agreement with the results described above regarding the significant effects of a family history of breast cancer, BMI, and post-menopausal status. However, in this analysis, striking life events were significantly associated with breast cancer (HR: 1.811, 95% CI: 1.021-3.212, *p* = 0.0423). The difference implied that we never really appreciated the depth and bitterness of the conflict in the relationship between breast cancer greater levels of stress, thus women with breast cancer were more likely to have greater levels of stress.

**Table 4:** Multivariate logistic regression analysis of the relationship of rank variables with breast cancer

Risk factor	OR	95% CI	*P* value
Age (years)			
20-29	1.000		
30-39	0.534	0.101 - 2.813	0.4591
40-49	0.588	0.114 - 3.030	0.5256
50-59	0.902	0.167 - 4.884	0.9051
60-70	1.187	0.201 -7 .017	0.8497
Menarche (years)			
<13	1.000		
13-14	1.409	0.848 - 2.340	0.1852
≥ 14	1.612	1.038 - 2.503	0.0336
BMI (kg/m^2^)			
<18.5	1.458	0.604-3.520	0.4019
18.5-24	1.000		
24-28	2.259	1.493-3.419	0.0001
≥ 28	3.823	1.782-8.200	0.0006
Stress			
0	1.000		
1-3	3.100	1.486 - 6.467	0.0026
4–6	2.925	1.471 - 5.817	0.0022
7–9	2.642	1.207 - 5.784	0.0151
Breast operation history.			
With	1.205	0.706 - 2.059	0.4938
Without	1.000		
Breast cancer history			
With	2.380	1.105 - 5.124	0.0267
Without	1.000		
OCP			
With	0.838	0.319 - 2.204	0.7204
Without	1.000		
Menopause			
Yes	0.450	0.252 - 0.803	0.0069
No	1.000		
Striking life events			
Yes	1.811	1.021 - 3.212	0.0423
No	1.000		

Abbreviations: OR, Odds Ratio; CI, confidence interval; OCP, use of oral contraceptive pills more than 1 year.

**Figure 2:**
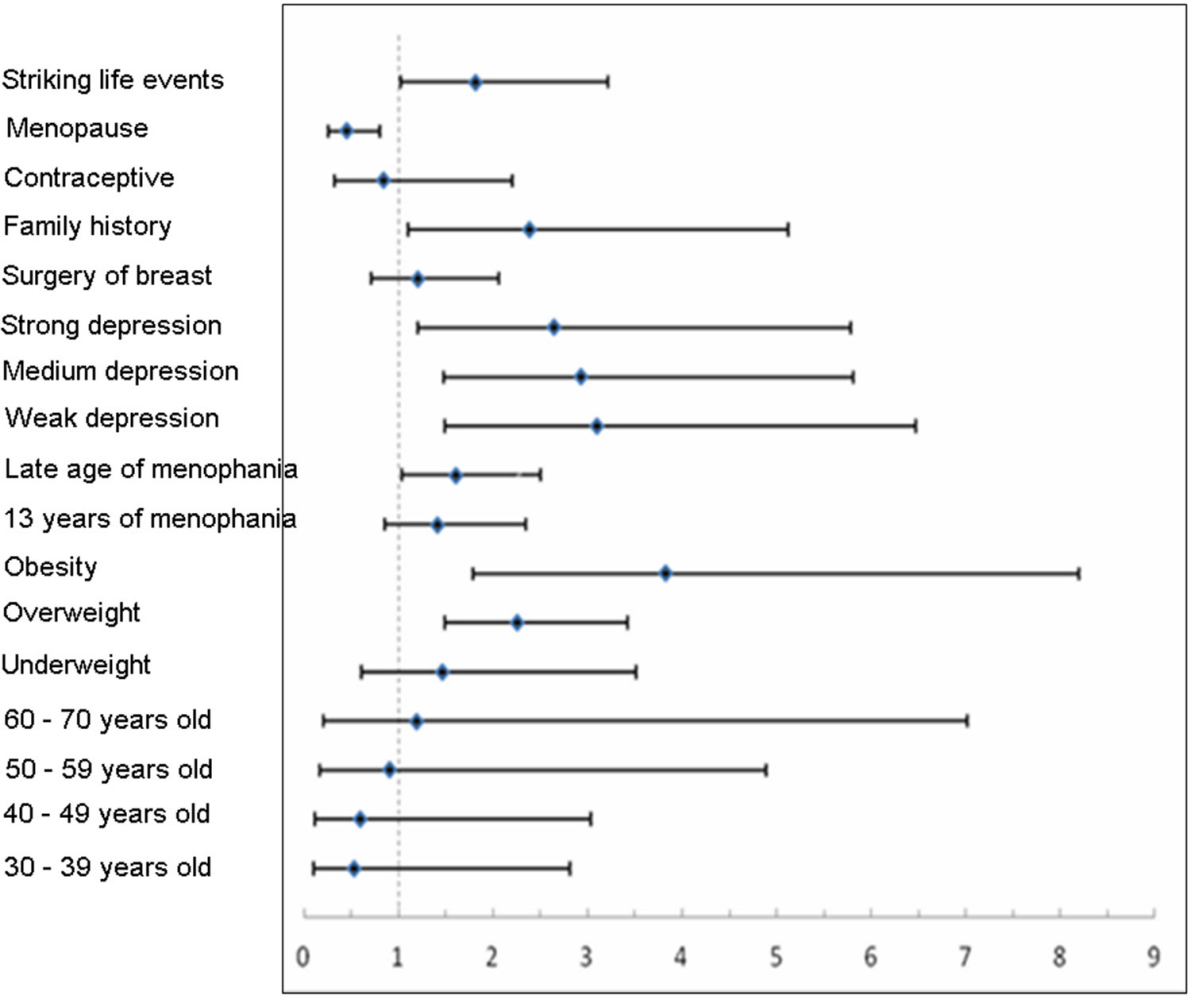
Forest plots of factors associated with breast cancer based on analysis of rank variables

Forest plots (Figure 2) show these same data graphically, and emphasize that only post-menopausal status was protective, and that a family history of breast cancer, elevated BMI, older age at menarche, increased stress, and striking life events were significantly harmful.

### Correlation of striking life events with breast cancer

Finally, we examined the correlation of striking life events and breast cancer with correction for age alone, with correction for multiple variables, and with correction for multiple variables in a subgroup analysis of women with late menarche (Table 5). The HR was marginally significant (*p* = 0.0480) in the age-adjusted analysis and marginally insignificant in the multivariable analysis (*p* = 0.0609). Subgroup analysis of women with late menarche also indicated a significant association of striking life events with breast cancer (HR: 1.811, 95% CI: 1.021-3.212, *p* = 0.0423).

**Table 5:** Correlation of striking life events and breast cancer with adjustment for age alone, multivariable adjustment, and multivariable adjustment in subgroup analysis of women with late menarche

	Age-adjusted	Multivariable-adjusted	Multivariable-adjusted, subgroup analysis
OR	1.726	1.716	1.811
95% CI	1.005 - 2.965	0.976 - 3.017	1.021 - 3.212
*P* value	0.0480	0.0609	0.0423

Abbreviations: OR, Odds Ratio; CI, confidence interval of estimated HR.

## DISCUSSION

Many recent studies have identified risk factors for breast cancer, but few studies have examined the association of life events with this cancer. In the Finnish Twin Cohort, a national modification of a standardized life event inventory was used, examining accumulation of life events and individual life events and placing emphasis on the 5 years. The multivariable adjusted hazard ratio for breast cancer per one-event increase in the total number of life events was 1.07, and divorce/separation, death of a husband, and death of a close relative or friend were all associated with increased risk of breast cancer, seemed a role for life events in breast cancer etiology through hormonal or other mechanisms [21]. In fact, the association of life events with breast cancer has become a contentious issue [28–30]. Kvikstad et al. performed a cohort study of 4491 cases with breast cancer and 44910 healthy controls in 1996 and demonstrated no significant effect of widowhood on breast cancer (HR: 1.13, 95% CI: 0.94-1.36) [25]. Surtees et al. also indicated no significant relationship between moderate-intensity striking life events and breast cancer [29]. On the other hand, both Geyer et al. and Peled et al. demonstrated that more than one striking life event increased the risk for breast cancer in a population of 255 cancer patients and 367 controls who were all younger than 45 years-old [30, 31]. Further, Kruk et al. compared 858 cases with breast cancer and 1085 controls and concluded that serious life events significantly increased the risk of breast cancer (HR: 5.07, 95% CI: 2.80-9.18) [28].

Interestingly, some previous studies of the effect of psychological or life events on breast cancer did not control for confounding variables [28]. Other studies controlled for different confounding variables, based on their own research or based on the literature review, and this could explain the conflicting conclusions. In present study, we used risk prediction factors in the model to control for confounding, and the risk prediction model was based on literature review and information from the largest population and case-control study of breast cancer in China, which enrolled 1202 cases (China National Eleventh Five Year Project). Thus, we more effectively controlled for confounding and also specifically focused on Chinese females, using our previous research as a foundation. Thus, the present study is the largest case-control study to investigate the association of striking life events with breast cancer in China.

Remarkably, the overall incidence of striking life events was only 17.4% among cases and 9% among controls. These data differ from the findings of several previous reports because these other studies also considered neighborhood disputes, poor relationships with a boss or colleague, and academic failure as striking or severe life events. Although these other items had a psychological impact, they were more akin to a bad mood or depressed state. Thus, we did not include these items, but only considered a depressed state in the risk assessment model.

Psychological factors that potentially increase the risk of breast cancer can be classified as chronic or acute. In our study, we evaluated striking life events that caused acute psychological trauma and emphasized the importance of the underlying psychological stimuli. In fact, previous research that analyzed specific types of stressful life events, such as major life events, concluded that there was a significant correlation between breast cancer and stressful life events [32]. Thus, studies of the effect of psychological factors on breast cancer need to consider the nature and severity of stressful events and whether these psychologically striking events have chronic or acute psychological effects.

There is still some dispute about how to define a “striking life event”. An earlier report used the Holmes-Rahe scale to classify striking life events [19]. This scale considers 43 different events and assigns an irritation score to each one, with the highest score for death of a spouse (100) and the lowest for a minor legal offense (11). Although these variables are distinct and the score is easily determined, individuals from different cultures will likely respond differently to these variables. Another report proposed modification and simplification of the self-rating scale of striking life events [33]. The different methods used to determine the total number of striking life events in an individual and the different definitions of major life events may be a reason for the different conclusions of previous studies. In present study, we evaluated striking life events of an individual with consideration of each subject’s own feelings; we allowed each subject to explain whether a striking life event occurred according to the subject’s own individual psychological experiences, and then classified the event. Although there was a certain amount of subjectivity, it avoided the ignorance of different psychological feeling which came from the divergence of the event nomenclature. Taking the same point of view, Chasan-Taber proposed that although a respondent’s self-evaluation was not always accurate, it did have good repeatability [34]. In the present study, we emphasized the intensity of life events, and classified these events into three major categories, using settings that were close to those used by Roberts et al [19]. In addition, the form of our questionnaire was simple and together with other factors, this would reduce bias due to excessive reinforcement of memory.

It is also important to define the time when a striking life event occurred. A previous study examined events that occurred one year before the diagnosis of breast cancer, and found that 82% women recalled at least one striking life event, and 56% women thought back the occurrence of major events [35]. In the present study, only 15% of women reported a striking life event in the 3 years before diagnosis of breast cancer. A major reason for the divergent opinions on the incidence and impact of striking life events may be the different criteria used to define such events. Moreover, some previous reports examined the effect of striking life events that occurred 5 or more years previously [18, 21]. In the present study, considering that an extended observation period may have reduced the clinical impact of striking life events, we focused on events that occurred in the three years before diagnosis in cases and three years before medical exams in controls. Examination of the effect of the duration between a striking life event and the diagnosis of breast cancer is a topic that requires further study.

The present study compared the characteristics of 265 cases with breast cancer and 265 controls. The most important finding was the positive association of striking life events with breast cancer (HR: 1.811, 95% CI: 1.021-3.212). Our comparison of the results of the univariate and multivariate analysis indicated that confounding factors were very important, and that striking life events lost statistical significance when there was no control for confounding. Adjustment for age alone led to a significant correlation (OR: 1.726, *p* = 0.0480) but adjustment for multiple factors led to no significant correlation (OR: 1.716, *p* = 0.0609), and the classification of variable grade after regression analysis led to a significant correlation (OR: 1.811, *p* = 0.0423). Thus, the association observed is limited to a certain category of life events.

The age of the highest incidence of breast cancer in China seemed younger than in Western countries in present study. In other report, in contrast, longitudinal age-specific rates were similar among all Asian countries and the United States with incidence rates rising continuously until age 80 years. The extrapolated estimates for the most recent cohorts in some Asian countries actually showed later ages at onset than in the United States [43].

In present study, the most important finding was the positive association between striking life events and breast cancer in multivariate analysis (HR: 1.811, *p* = 0.0423). However, the HR for this association was lower in the univariate analysis, indicating that confounding factors weakened the relationship. This may be the reason for the conflicting results in previous reports. Remarkably, adjustment for age alone led to a significant association of striking life events with breast cancer (HR: 1.726, *p* = 0.0480). Although there was no such relationship in multivariate regression analysis (HR: 1.716, *p* = 0.0609), it was a participant again in regression analysis after classification of variable (OR: 1.811, *p* = 0.0423). This demonstrates that the relationship of striking life events with breast cancer rises to statistical significance only when considering the influence of certain confounding variables.

Our analysis of the different types of striking life events indicated that emotional injury from family events (such as the death or illness of a relative) was the most common, followed by marriage-related issues, and then the economic issues (such as unemployment or major property damage). The incidence of each different class of striking events was similar in cases and controls. However, the overall number of striking life events was greater in our cases than controls. Moreover, our multivariate analysis indicated that striking life events had a more modest effect in increasing the risk of breast cancer that reported by Kruk et al. (HR: 5.09) and Chen et al. (HR: 11.64), but similar to our previous meta-analysis [14, 36]. These results imply that usage of risk factors specific for Chinese individuals is needed to adequately control for confounding, but this must be confirmed by a future large prospective study.

In addition, the present study indicated associations of breast cancer with menopausal status, family history of breast cancer, and BMI. Our finding that early menopause was a protective factor is in concert with other reports [37] and our finding that a family history of breast cancer and a higher BMI increased the risk of breast cancer is also consistent with previous reports [38]. More importantly, regarding our finding of the associations of late menarche and greater life pressure with breast cancer, the fact that positive correlations only occurred in subgroup analysis demonstrated that the connection of less dangerous factors were often assume their aspect under strict control of confounding variables. Our findings regarding the protective relevance to breast cancer of early menarchy are consistent with our previous study but in contrast to other reports; whether it is the result of the special characteristics of Chinese women, or the result of the sample size, would require further follow-up cohort study.

A possible direction of bias in present study is that researchers administered the questionnaires to most breast cancer patients, and these patients were given ample time to explain the nature of different striking life events, but the controls completed the questionnaires by themselves with limited time. This may have contributed to the greater number of striking life events in cases than controls. Second, the time from diagnosis to data collection may have been shorter in our cases than controls. Previous studies used survey intervals of 2 years or 7.7 months, and both groups of researchers noted that a longer interval would lead to greater recall bias [35, 36]. Meanwhile, the cases are aware of their diagnosis at the time of administering the questionnaire. Considering the inevitability of recall bias, we selected recent surgery patients as cases. Third, the magnitude of bias may be manifested in cases selection because all selected cases needed further treatment after surgery, so we did not include patients with early-stage breast cancer. Similarly, cases with breast cancer *in situ* (stage 0) would not be included because the most recent version of breast cancer staging considers breast cancer *in situ* as a precancerous lesion.

Some additional limitations of this study should be noted. First, we did not analyze individual personality characteristics, different methods of coping, or the use of social support systems. Some previous reports found that a tendency for anger or other negative behaviors was an important and independent risk factor for breast cancer, and proposed that venting or suppression of anger may have been a key factor [39, 40]. Second, in order to reduce the effects of bias, a prospective study is needed to verify the relationship between striking life events and breast cancer. Such a prospective cohort study should be performed in the future. In addition, the effects of psychologically striking events on the immune system or endocrine system are mostly at the level of hypotheses, and this topic must also be specifically investigated.

## MATERIALS AND METHODS

### Study population

This 1:1 age-matched case-control study was carried out in Peking Union Medical College Hospital of Peking Union Medical College from January 2011 to July 2012. Each case was age-matched with a control whose age differed by less than 3 years. The cases were 26 to 69 years-old (mean: 47.2 years, standard deviation: 8.6), had pathological diagnoses of invasive breast cancer, had no psychiatric illnesses, and received radical breast resection from May 2011 to Dec 2011 in the Department of Breast Disease. Each case had full understanding of her disease and was able to independently complete the survey form. A life event inventory was included in the questionnaire. The controls were healthy individuals selected from the Physical Examination Center or Department of Breast Disease in Peking Union Medical College. These women were 27 to 71 years-old (mean: 46.5 years, standard deviation: 8.5), did not have invasive breast cancer, and had no psychiatric illnesses. All methods were carried out in accordance with relevant guidelines and regulations. All experimental protocols and data collection were approved by the Peking Union Medical College Hospital Medical Ethics Committee (No. S-406). The informed consent was obtained from all subjects. Sample size calculations were carried out before starting the study according to the previous formula [41].

### Questionnaire content

The present study focused on the effect of psychologically striking events, including illness or death of relatives, divorce or separation, emotional trauma, unemployment, and significant loss of property. Each patient self-reported the psychological effect of these different stressors. Thus, the death of a relative may have led to depression and even forced some individuals into early retirement due to the psychological stress. However, other individuals may have experienced no psychological trauma following the death of a relative if they were not living together or if the relative was elderly and had a natural death. A series of previous studies were as referenced methodological issues to form the final questionnaire content [5, 9, 11, 14, 17, 21].

It was also necessary to consider possible risk factors of breast cancer outside the psychologically striking events to avoid confounding errors. However, the risk factors for breast cancer vary in different countries, and there appear to be unique risk factors for Chinese women. Thus, we considered factors based on the previous literature and our own previous report, the largest survey of Chinese women, which was funded by the Chinese 11th Five-Year Research Plan (No. 2006BAI02A09) [27].

Thus, we considered height, weight, body mass index (BMI), age, age at menarche, level of stress (scored 0 to 9), history of breast biopsy (not including the biopsy prior to radical resection), presence of a family member (parent, child, brother, or sister) with breast cancer, use of oral contraceptives for more than 1 year, and post-menopausal status (defined by bilateral ovarian resection, no menstruation in the previous year, or age above 60 years-old). Although previous studies of Chinese women did not report associations of childbearing and lactation with breast cancer, other research has classified these two items as risk factors, so our present survey also considered them.

### Assessment of striking events

Among cases and controls, all information was collected within 12 months of the diagnosis of breast cancer (cases) or during an interview (controls). The presence of striking life events was assessed by asking several questions: “In the 3 years before your diagnosis of breast cancer, were there any mentally or spiritually stressful events?” “Please describe which of the listed items were not psychological blows to you.” “Was your specific psychological blow excluded the listed items?” “If you would rather not describe the specific psychological blow, please classify the event into 1 of 3 categories.”

Additional information was collected from controls and cases including age, height, weight, age at menarche, stress, history of surgery, family history, menopausal status, use of oral contraceptives, and striking events within the previous 3 years.

### Assessment of covariates

Among cases, a single investigator administered the questionnaire to most patients (82%) to reduce the possibility of judgment bias by the researcher. Other cases required help for completion of forms, and this was strictly screened by the same researcher. The rejection rate was 37%. Among controls, information was from a large database, and each case was age-matched (maximum difference of 3 years) with a control. These data were collected following a short-term training of doctors and nurses by use of unified standards and methods to reduce sampling bias in information acquisition. Moreover, a double entry method was used for data input; one method used Epidate 3.0 software in which the variable was assigned a value and the other used a breast cancer risk prediction model (Figure 1) and automatic conversion into MS Excel format. The checking and cleansing of data also employed pair-wise alignment.

### Statistical analysis

Results of the analyses were first adjusted only for age (continuous variable). The next set of analyses examined whether the age-adjusted estimates were confounded by menarche (<13 years, 13–14, or >14 years), body mass index (< 18.5 kg/m^2^, 18.5–24, 24–28, or ≥ 28 kg/m^2^), stress scores (0, 1-3, 3-6, or 7-9), breast operation history (with or without, but does not include a biopsy before radical surgery for breast cancer), breast cancer history (with or without), use of oral contraceptives (never or ever), and post-menopausal (yes or no). Further adjustment was made with correction for multiple variables. Finally, in the analyses of individual life events, we also adjusted with correction for multiple variables in a subgroup analysis of women with late menarche. We retained subjects with missing values for any of the covariates by including them in the reference category for the relevant covariate.

All calculations were performed using SAS 9.0 statistical software. The presence of breast cancer was the dependent variable and the presence of striking events and other factors (age, BMI, age at menarche, stress scores, breast operation history, breast cancer history, contraceptive use, and menopause) were the independent variables. The chi square test was used to assess the significance of differences between the 2 groups. Then, logistic regression analysis was used to assess the association of psychologically striking events and each categorical variables with the occurrence of breast cancer, respectively. Continuous variable was changed to rank variable, and striking life events were ranked into with or without events. Multiple logistic regression analysis was used to assess the association of psychologically striking events and other variables with the occurrence of breast cancer. The odds ratios (ORs) and 95% confidence intervals (CI) were calculated. A result was considered statistically significant if the *p*-value was less than 0.05.

## CONCLUSION

The present study of Chinese women indicated a positive relationship between striking life events and breast cancer. Breast cancer was also associated with obesity, family history of breast cancer, late menarche, late menopause, and overall stress. Specially, the association is not universal but applies to a certain impact of life events.

### Use of human subjects

We have all necessary consent from any patients involved in the study, including consent to participate in the study where appropriate. We confirmed that all experiments were performed in accordance with relevant guidelines and regulations. The present study and data collection were approved by the Peking Union Medical College Hospital Medical Ethics Committee (No. S-406).

## Abbreviations

BMI: body mass index
CI: confidence intervals
ORs: odds ratios
